# Design of a Fatigue Detection System for High-Speed Trains Based on Driver Vigilance Using a Wireless Wearable EEG

**DOI:** 10.3390/s17030486

**Published:** 2017-03-01

**Authors:** Xiaoliang Zhang, Jiali Li, Yugang Liu, Zutao Zhang, Zhuojun Wang, Dianyuan Luo, Xiang Zhou, Miankuan Zhu, Waleed Salman, Guangdi Hu, Chunbai Wang

**Affiliations:** 1 School of Information Science and Technical, Southwest Jiaotong University, Chengdu 610031, China; zxl627905@163.com (X.Z.); luodianyuan@my.swjtu.edu.cn (D.L.); zx871809141@gmail.com (X.Z.); zhumiankuan@126.com (M.Z.); 2 School of Transportation and Logistics, Southwest Jiaotong University, Chengdu 610031, China; lijiali@my.swjtu.edu.cn (J.L.); wangzhuojun163@126.com (Z.W.); 3 School of Mechanical Engineering, Southwest Jiaotong University, Chengdu 610031, China; zzt.swjtu.edu.cn (Z.Z.); wldsalman6@gmail.com (W.S.); ghu@home.swjtu.edu.cn (G.H.); 4 Department of Industrial and Manufacturing Systems Engineering, Iowa State University, Ames, IA 50011, USA; chbwang@iastate.edu

**Keywords:** high-speed train safety, vigilance detection, wireless wearable, brain-computer interface, fatigue detection system

## Abstract

The vigilance of the driver is important for railway safety, despite not being included in the safety management system (SMS) for high-speed train safety. In this paper, a novel fatigue detection system for high-speed train safety based on monitoring train driver vigilance using a wireless wearable electroencephalograph (EEG) is presented. This system is designed to detect whether the driver is drowsiness. The proposed system consists of three main parts: (1) a wireless wearable EEG collection; (2) train driver vigilance detection; and (3) early warning device for train driver. In the first part, an 8-channel wireless wearable brain-computer interface (BCI) device acquires the locomotive driver’s brain EEG signal comfortably under high-speed train-driving conditions. The recorded data are transmitted to a personal computer (PC) via Bluetooth. In the second step, a support vector machine (SVM) classification algorithm is implemented to determine the vigilance level using the Fast Fourier transform (FFT) to extract the EEG power spectrum density (PSD). In addition, an early warning device begins to work if fatigue is detected. The simulation and test results demonstrate the feasibility of the proposed fatigue detection system for high-speed train safety.

## 1. Introduction

From 1 April 1997 to 18 April 2007, the railway transportation in China experienced six great “improvements” in train speed. The speed of Chinese high-speed trains has been raised to 200 km/h, and accordingly, the Chinese high-speed train technology is the worldwide leader. Since the “7.23” Yong-Tai-Wen railway accident (which occurred on 23 July 2011), the most serious railway accident in Chinese railway history, high-speed train accident prevention in China has been changed from passive control modes to active ones [1]. According to the accident investigation report, the causes of the accident were associated with the design, approval, and use of the TCC (the Train Control Centre). Little attention is paid to the important part of the high-speed train Safety Management System (SMS), namely, the “emergency management” during an accident. The literature shows that it is the SMS, such as organization, communication-team work, system design, and quality of procedures and perception, that mainly affects the high-speed train in terms of safety. However, the train drivers, signallers, and controllers are also important to railway safety. Especially in case of an emergency, the driver’s performance is a major contributor to incidents and accidents [2,3,4]. It is crucial to analyse the contribution of the driver’s performance to railway safety and accidents.

### 1.1. Train Safety and Accidents Analysis

The safety of the railway depends on several factors, including the safety culture, communication-team work, system design, quality of procedures, perception, information system, shift pattern and workload. Numerous studies have been performed to investigate the causes of accidents on the railway system. Table 1 summarizes the distribution of occurrence types of 78 train accidents reported in the UK [5]. The top 3 occurrence categories of railway accidents are human failure (HF) to collision, derailment, and level crossing occurrence. As shown in Table 2, nearly 39.74% (31 out of 78) of the train accidents fell into these three categories. This shows that Human failure is a major contributor to railway accidents, which mainly affect the railway system in terms of safety. In Europe, literature by Evans [6] shows that at least 75% of the fatal railway accidents between 1990 and 2009 were due to human error, e.g., exceeding speed, signal passed at danger, signalling/dispatching error, etc. Table 3 shows the results of railway-performance shaping factors (R-PSFs) of train safety in serious accident analysis, where 10 factors responsible for railway accidents are listed. In Table 3, three red boxes show the railway-performance shaping factors as distraction (loss of concentration or vigilance), fatigue (shift pattern) and workload (time pressure and stress). The proportions of these three factors were 15.76%, 5.76% and 3.73%, respectively. This shows that driver fatigue has always been an important factor in transportation accidents, particularly for high-speed train drivers who work in a monotonous and high-concentration-demanding circumstance for many work hours.

### 1.2. Driver Vigilance Detection Technologies

Studies of train driver fatigue have shown that critical incidents are more likely to occur at certain times of the day and at certain periods within a duty [7,8,9]. Fatigue and vigilance, the first two of the highest-ranking topics, were found to be critical problems of railroad operational safety in 2006 [10]. Especially for high-speed train safety, the vigilance of the driver is a major factor in maintaining basic awareness of oncoming traffic [11].

The Chinese traditional train driver alarm system requires the driver to pedal at least once every 30 s [12]. Otherwise, the control system of the train assumes he is not alert and sends an alarm to the driver. If the driver still fails to step on the pedal after 7 s, the train will automatically brake to a stop for the safety of the passengers. Although it is a safe strategy guarding against the declination of driver vigilance, an undesired braking action will result in time loss and disruption to railway schedules. Moreover, to improve the efficiency of railway operation and personnel utilization. Most countries have adopted a single-driver operating system [13]. If the driver is drowsy, no second driver can operate the train. After the train brakes, no other vigilant driver can restart the train. Therefore, it is necessary to detect and relieve the fatigue of drivers for high-speed train safety in the course of trains running on schedule.

Technologies to detect the vehicle driver’s vigilance have been rapidly developed by various vehicle manufacturers, for example, in the applications of a drowsy-driver-detection system for Volvo trucks [14]. In the literature [15], authors have evaluated the use of fatigue detection technologies in a fatigue risk management system for the transport industry. They proposed a set of evaluative and operational criteria for organizations and regulators to contemplate the use of these technologies. However, in the railway transportation field, most studies focus only on the management of driver shift system procedures, policies, and regulations of related companies or the railroad administration to avoid incidents [16,17]. In high-speed trains, the driver’s vigilance can be the main factor that affects the railway system in terms of safety, especially in the case of emergency.

The driver’s vigilance detection technology can be divided into three main categories: (1) vehicle-behaviour-based technology; (2) driver-behaviour-based systems; and (3) driver-physiological-signal-based algorithms [18,19,20,21,22]. The first category is not suitable for trains because they use a track [23]. The second category analyses the changes of driver behaviour, such as eye tracking, yawning, percent eye closure (PERCLOS), blink frequency, nodding frequency, facial position, and inclination of the driver’s head [24,25,26,27,28]. Recent progress in machine vision research and advances in computer hardware technologies have made it possible to measure eyelid movement, face expression and head pose using video cameras. Usually, a recorded video is used to analyse and classify the vigilance level of the driver. The quality of video images is susceptible to or dependent on environmental and driver conditions, such as light conditions, the glass of driver, etc. Furthermore, false estimation can also be caused by the variability of the driver’s behaviours, such as sleeping with open eyes.

The last category is the driver-physiological-signal-based algorithms for driver vigilance detection, using electrocardiosignal (ECG), electrooculography (EOG), electroencephalographic (EEG), or Heart Rate Variability (HRV) [29,30,31,32,33]. These systems are more reliable because physiological signs of drowsiness are well known and rather similar. The EEG signal is always regarded as a “gold standard” of vigilance detection. Proven techniques of signals processing, such as date compressing, de-noising, feature extraction and classification, make it be possible to cope with the EEG single. Principal Component Analysis (PCA), independent component analysis (ICA), sparse representation and compressed sensing and so on are widely applied to EEG detection [2,34,35,36,37,38]. The literature shows that a typical sleep stage can be divided into non-rapid-eye-movement (NREM) sleep and rapid-eye-movement (REM) sleep based on the EEG signal [39]. NREM sleep is further subdivided into stages 1 to 3. The first stage, which is the transition period from alertness to sleep, is considered to be the drowsy stage in this paper. In this stage, the increasing power of theta (4–8 Hz) and alpha (8–14 Hz) waves is observed by Parikh [40], as well as the decreasing power of beta (14–34 Hz) waves at the area of occipital sites (O1 and O2). The difficulties of the driver-physiological-signal-based measures lie in how to obtain EEG signal recordings comfortably under driving conditions and classify the driver vigilance with so many EEG signals. Nevertheless, the physiological signal measures are believed to be accurate, valid, and objective in determining driver vigilance. Currently, there are two front-ends for EEG signal collection: dry electrodes and wet electrodes. A wet electrode tests with a smearing conducting solution. Thus, it can be used only under special circumstances. Compared with the wet electrode, there is no limit of dielectric or environment for the dry electrode [41,42,43,44,45]. Table 4 shows a survey and comparison of the existing systems used for driver vigilance monitoring.

Despite the success of the existing approaches/systems for driver vigilance detection of active vehicle safety, until now, few researchers have investigated high-speed train safety deeply and systematically based on train driver’s vigilance detection. In this paper, a fatigue warning system for high-speed train safety is introduced based on train driver vigilance detection using wireless wearable EEG collection technology. An 8-channel wireless wearable brain–computer interface (BCI) hardware system is designed to collect the train driver’s EEG signal comfortably. A support vector machine (SVM) classification algorithm with the Fast Fourier transform (FFT) for extraction of the EEG power spectrum density (PSD) is implemented for the high-speed train driver’s EEG signal to determine the vigilance level. The proposed system is the first scheme to design a novel fatigue detection system for high-speed train safety.

The rest of this paper is organized as follows: in Section 2, the general system architecture of the proposed fatigue detection system is presented. Section 3 focuses on the wireless wearable EEG collection system for the train driver. Train Driver vigilance detection is developed in Section 4, and the early warning system is described in Section 5. The experiments and analysis are reported in Section 6. Finally, some conclusions are provided in Section 7.

## 2. System Architecture

**Figure 1:**
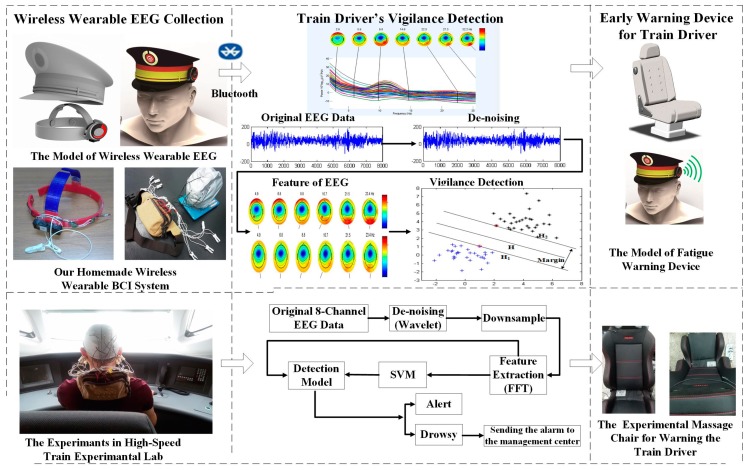
Flowchart of the proposed high-speed train driver fatigue relief system.

The general architecture of the fatigue detection system, as illustrated in Figure 1, has three major steps: (1) the wireless wearable EEG collection system; (2) the train driver vigilance detection system; and (3) the early warning device.

In the first step, an eight-channel wireless wearable BCI system collects the driver’s EEG signal and transmits the recorded data to a PC/FPGA/DSP via a Bluetooth interface. As shown on the left of Figure 1, a wireless wearable EEG collection system embedded in the driver’s cap is designed. The BCI system consists of eight stainless steel dry electrodes. It incorporates the use of a wireless and wearable EEG device to conveniently record EEG signals from the head regions of the train driver.

The second step is train driver vigilance detection using SVM with the FFT to extract the PSD of the EEG signal. As shown in the middle block of Figure 1, after the original EEG signal is collected from the train driver’s head, a wavelet de-noising algorithm is implemented to remove interference, such as blinking (<5 Hz) and electromyography (>30 Hz). Then, the PSD is extracted as the feature of each state (alert/drowsy) using the FFT. Finally, an SVM algorithm is proposed to establish a vigilance detection model based on the training data. In this paper, two vigilance levels are defined: alert and drowsy. In the experiment and simulation, a personal computer is used to process the simulation data. The improved versions will be completed in the experiment using DSP or FPGA.

In the last step, as shown in the right part of Figure 1, an early warning system is designed. When fatigue is detected, a massage chair begins to work to warn the high-speed train driver. At the same time, the early warning system messages about the high-speed train driver’s situation is sent to the TCC.

## 3. Wireless Wearable EEG System for High-Speed Train Drivers

**Figure 2:**
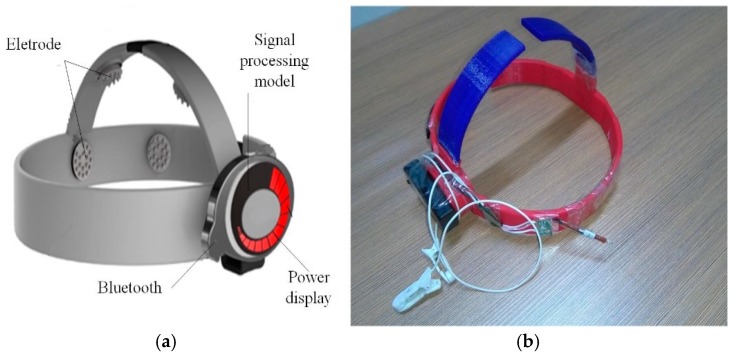
8-channel wireless wearable BCI system. (**a**) BCI system model; (**b**) Homemade BCI system.

**Figure 3:**
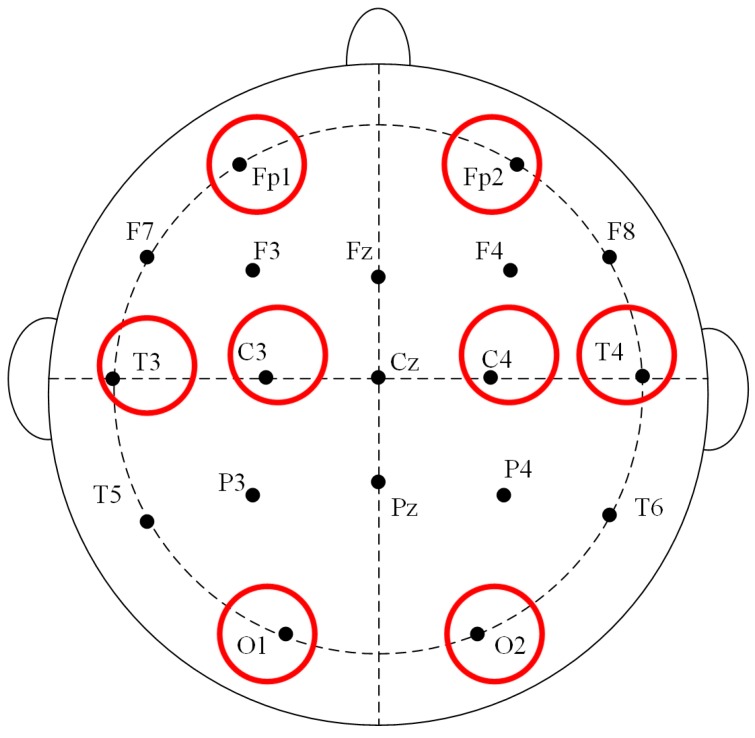
10–20 electrode system.

**Figure 4:**
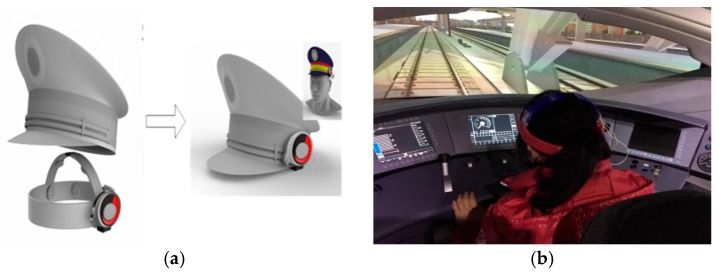
Usage of the BCI system. (**a**) diagram of usage; (**b**) Field usage.

EEG data are highly important to our train driver vigilance detection. To collect the EEG signal of the driver, an eight-channel wireless wearable BCI system is designed as shown in Figure 2. The electrodes, which are a type of sensor for EEG acquisition, are placed on the pre-frontal (Fp1, Fp2), temporal (T3, T4), posterior occipital (O1, O2), and central (C3, C4) regions of the cerebrum according to the 10–20 electrode system in Figure 3. The signal process model stores the collected signal. The Bluetooth in front of the signal-processing model transmits the data to a PC. The power display outside the signal-processing model informs the user of how much power remains. Figure 4 shows usage of the BCI system. By referencing the appearance of the train driver’s hat, the driver vigilance detection device can be easily embedded into the cap, and it hardly affects the driver’s operation.

**Figure 5:**
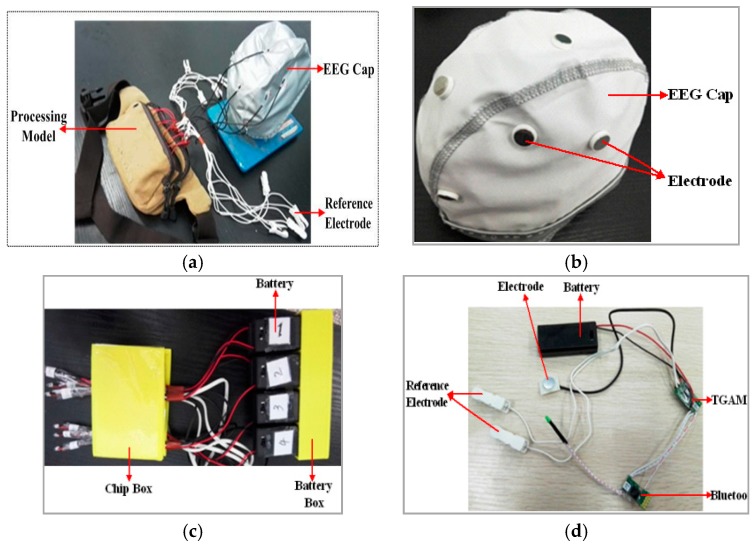
Fabrication of our proposed wireless wearable BCI system for experiments. (**a**) Wireless wearable BCI system for train driver; (**b**) Electrode cap for experiments; (**c**) Single-channel wireless wearable EEG collection model; (**d**) The processing model for experiments.

A set of high-comfort wireless wearable BCI equipment for testing and simulation in the lab has been made. The basic scheme of our homemade EEG-based BCI system is as proposed in Figure 5. The BCI system consists of an EEG collection cap, reference electrodes, and processing model (see Figure 5a). In Figure 5b, the positions of eight dry electrodes installed in the EEG collection cap correspond to the cerebrum areas. The installation positions have close relationships with driver vigilance levels detection. In the homemade wireless wearable BCI system, the EEG collection cap is able to comfortably collect the train driver’s brain signal data for testing and simulation in the lab. There are eight single-channel EEG collection models. As shown in Figure 5c, for a single model, the structure has a five-part structure: (1) a stainless steel dry electrode; (2) a TGAM model (the chip used to process the EEG signal); (3) a Bluetooth model; (4) a reference electrode; and (5) a battery model. The EEG signal is obtained by the stainless steel dry electrodes first and then amplified and filtered by the think gear basic module (TGAM) model with hardware filtering of 3 Hz to 100 Hz and a sampling rate of 512 Hz. Next, the EEG signal is transmitted to the PC via Bluetooth. The reference electrode provides the reference potential for the stainless steel dry electrode. The system is powered by eight 3-V DC batteries. Figure 5d shows the processing model, including the TGMA, Bluetooth, and batteries installed into some boxes, which were designed by *SolidWorks* software and manufactured by a 3D printer. Finally, all chips of the TGAM, Bluetooth and batteries are arranged into a bag, as shown in Figure 5a, to make the equipment more wearable for train driver experiments.

To validate the efficiency of our homemade wearable BCI system in data acquisition accuracy, a 64-channel EEG provided by Brain Products (BP, Gilching, Germany) was used. 

**Figure 6:**
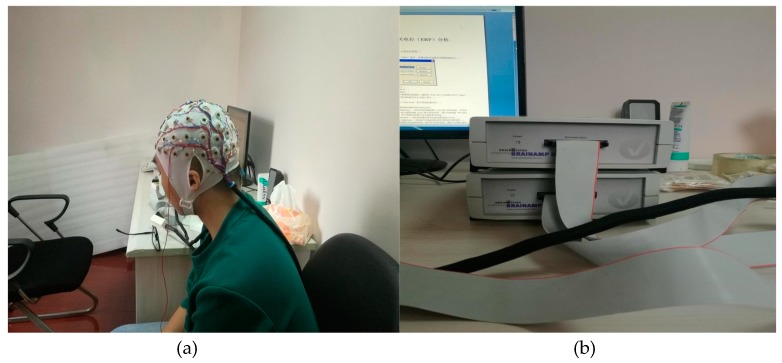
BP equipment. (**a**) BP collection cap; (**b**) BP signal processing box.

As shown in Figure 6, the commercial BP system consists of a Brain Cap, a Brain Amp, and the Recorder Analysis Software.

## 4. Train Driver Vigilance Detection

After a high-speed train driver’s EEG signal is obtained, data processing begins in the next steps, described as follows:

### 4.1. Data Preprocessing

**Figure 7:**
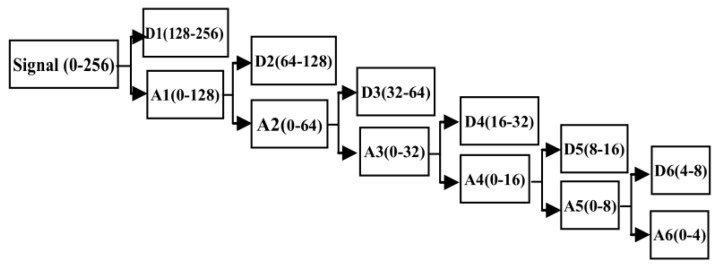
Decomposition space tree.

**Figure 8:**
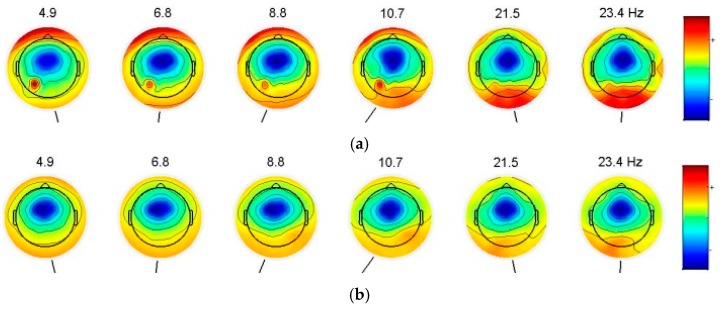
Power scalp topographies of various frequency components. (**a**) power scalp topographies in alertness; (**b**) power scalp topographies in drowsiness.

The purpose of this step is to extract the features of alert and drowsy EEG signals of train drivers by removing various interference. In this paper, a wavelet de-noising method is introduced because of its multi-resolution capability, which is appropriate for the non-stationary EEG signal. A six-layer decomposition of the db5 wavelet is implemented for the original EEG signal to extract theta, alpha and beta rhythms. The 6-layer decomposition is implemented to get the sub-band wavelet detail coefficients (**D*_i_***, *i* = 1, 2, 3, 4, 5, 6) and approximation coefficients (**A*_i_***, *i* = 1, 2, 3, 4, 5, 6). The decomposition space tree and frequency range are shown in Figure 7. By reconstructing the decomposition coefficients of **D_3_**, **D_4_**, **D_5_**, and **D_6_**, the useful EEG signal is extracted, and various low-frequency and high-frequency interferences are removed. Then, the PSD is adopted as the train driver’s EEG feature to distinguish two states. The significant differences of the power scalp topographies of various frequency components in the states of (a) alert and (b) drowsy are shown in Figure 8. 

In the alert case, the lower-frequency components are found in the area of the forehead, and the higher-frequency components are distributed in the occipital area. In the case of drowsiness, low-frequency components and high-frequency components in the forehead and occipital areas are approximately uniform. Different states between the occipital and forehead areas are distinguished by using the PSD of different frequency components. Under the assumption that the driver’s state at time t remains identical to that within the previous r seconds, the feature of the EEG signal at time t is calculated as the average PSD of the previous **s** seconds and t seconds. The PSD of each second is calculated using FFT and is subsequently converted into a logarithmic scale. Next, a Hanning Window is used to extract the PSD of theta, alpha, beta rhythms as the feature of drowsiness and alertness.

### 4.2. Vigilance Detection Based on SVM Classification

Developed from statistical learning theory (SLT), the SVM maps the input data to a high-dimensional feature space and constructs a linear optimal classification hyperplane. It addresses a nonlinear EEG data classification problem. The theoretical analyses of linear and nonlinear SVMs are described as follows.

**Figure 9:**
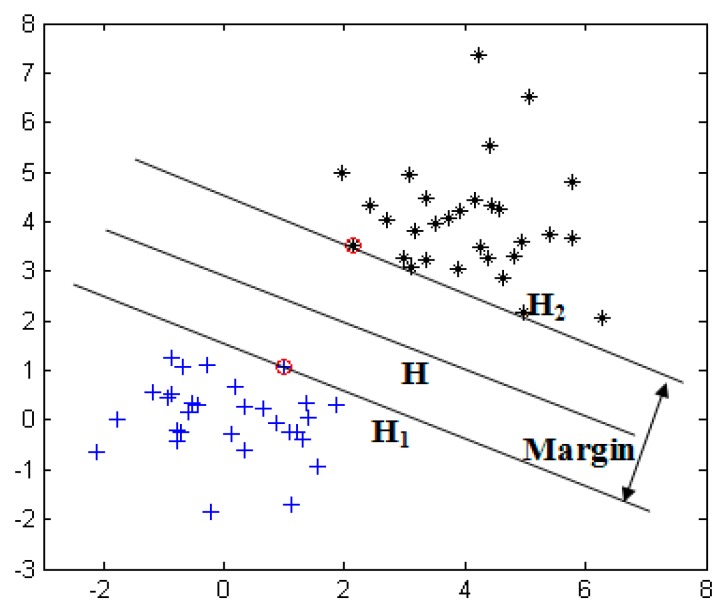
Linear SVM classification.

As shown in Figure 9, the linear SVM separates two classes of sample points, denoted by ‘*’ and ‘+,’ using the optimal hyperplane **H** and maximizes the margin, which is the distance between **H_1_** and **H_2_** (the lines pass through the nearest point away from **H**). Assume that the training data set is **T** = {(**x_1_**, **y_1_**), (**x_2_**, **y_2_**), …, (**x*_m_***, **y*_m_***)} (**x*_i_*∈***R**^n^***, **y*_i_*∈**{1, −1}), where **x_i_** is the training driver’s EEG data, **y*_i_*** is the label of the data class, ***m*** is the number of training data, and ***n*** is the dimension of **x*_i_***. In this paper, **y*_i_*** = 1 represents the class of **x*_i_*** that is alert, and **y*_i_*** = −1 represents the class of **x*_i_*** that is drowsy. For the linear classification case, the training data can be separated by the hyperplane **H** in (1):

(1) w⋅x+b=0

where **w** is the weight vector and **b** is the bias vector.

Therefore, **H_1_** and **H_2_** can be represented by (2):

(2) $$\{\begin{array}{cc} w\cdot x+b=+1 & H_{1}\\ w\cdot x+b=-1 & H_{2} \end{array}$$

Thus, the margin in Figure 5 is represented as $\frac{2}{\left\|w\right\|}$ and calculated using (1) and (2).

Ideally, all sample points can be separated by **H** and are located outside the margin. In reality, because of the noise or the complexity of the training data, some sample points are inside the margin, which makes the linearly separable SVM invalid. Therefore, a positive slack variable ***ξ*** is introduced to transform it to a linearly separable approximate situation, which is defined as (3):

(3) $$\{\begin{array}{cc} w\cdot x_{i}+b\geq +1-\xi_{i} & {\mathrm{if}\;y}_{i}=+1\\ w\cdot x_{i}+b\leq -1+\xi_{i} & {\mathrm{if}\;y}_{i}=-1 \end{array}$$

where ***ξ_i_*** is introduced to measure the deviation between **y*_i_*** and **w**·**x*_i_*** = **b**. If 0 < ***ξ_i_*** < 1, **x_i_** is inside the margin and is correctly classified. If ***ξ_i_*** > 1, **x_i_** is inside the margin but falsely classified. Finally, (3) can be simplified as (4):

(4) $$y_{i}(w\cdot x_{i}+b)\geq 1-\xi_{i},\;i=1,2,\ldots m.$$

The classification hyperplane is represented as w^*^·**x** = b^*^ = 0, and the optimal classification function is calculated using (5):

(5) $$f(x)=\mathrm{sgn}(w^{*}\cdot x+b^{*})=\mathrm{sgn}\{\sum_{i=1}^{m}y_{i}a_{i}(x_{i}\cdot x)+b\}$$

To approximate the nonlinear separable situation, the input **x** is first mapped to a higher-dimensional space via a nonlinear mapping ϕ(x). Thus, the approximate nonlinear separable SVM classification function can be rewritten as (6):

(6) $$f(x)=\mathrm{sgn}\{\sum_{i=1}^{m}y_{i}a_{i}\kappa (x_{i},x)+b\}$$

where $\kappa (x_{i},x)=(\phi (x_{i})\cdot \phi (x))$ is called a kernel function. Any function that satisfies the Mercer theorem can be used as a kernel function. Some common kernel functions are as follows:

Linear kernel: $\kappa (x,x_{i})=(x\cdot x_{i}).$
Polynomial kernel: $\kappa (x,x_{i})={\left((x\cdot x_{i})+c\right)}^{d},c\geq 0.$
RBF kernel: $\kappa (x,x_{i})=\exp(-g{\left\|x-x_{i}\right\|}^{2}),g\geq 0.$

After determining the vigilance level using (6), the train driver’s vigilance level is sent to the designed massage chair via Bluetooth. Then, the massage chair begins to work. At the same time, the train driver’s vigilance system sends an alarm signal to the train driver, and the alarm signal will be uploaded to the management operation centre.

## 5. Early Warning System

**Figure 10:**
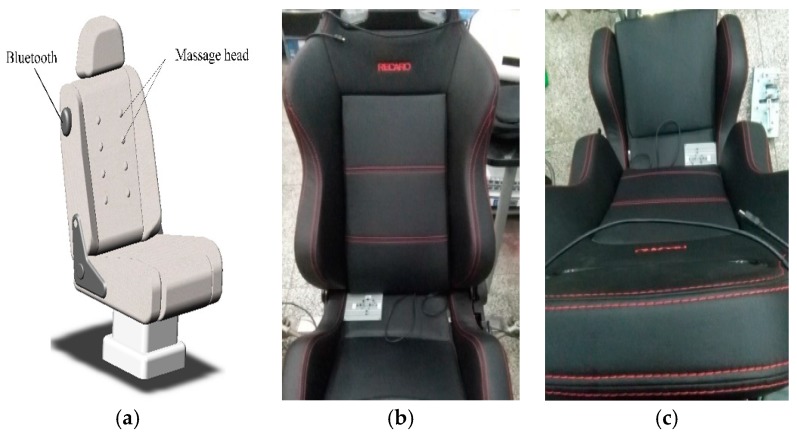
High-speed train driver’s fatigue warning device. (**a**) The model of vibrate chair; (**b**) Experimental massage chair in the test; (**c**) Experimental massage chair in the test.

In this paper, an early warning system is designed. If fatigue is detected, a massage chair can be used to continuously warn the high-speed train driver. As shown in Figure 10, considering the existing massage chair in the market and the appearance of the train driver’s chair, a fatigue-warning device was designed and modelled using *SolidWorks*. 

Eight massage heads with high mechanical vibrational frequency, which are evenly distributed on the chair back, can rotate to massage the back of the driver. The Bluetooth located lateral of the chair is used to receive the information about the driver’s vigilance level (drowsy or alert). If the driver is detected drowsy, the massage chair begins to work. At the same time, the early warning system is designed to send a warning message about the high-speed train driver’s vigilance level.

## 6. Experiments and Analysis

The goal of this section is to demonstrate experimentally and scientifically the validity of the proposed fatigue detection system for high-speed trains based on train driver vigilance using the wireless wearable EEG. It is extremely important to affect the high-speed train safety and avoid railway incidents and accidents. An experimental environment to evaluate the proposed system’s performance has been implemented in State Key Laboratory of Traction Power, Southwest Jiaotong University in China.

**Figure 11:**
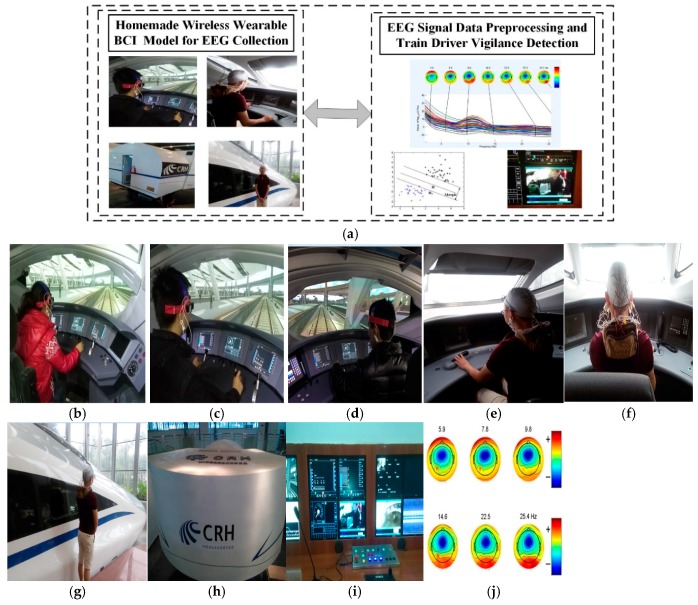
Experimental environment. (**a**) Experimental prototype; (**b**) EEG collection experiment; (**c**) EEG collection experiment; (**d**) EEG collection experiment; (**e**) EEG collection experiment; (**f**) EEG collection experiment; (**g**) High-speed train experimental simulator; (**h**) CRH high-speed train simulation cab; (**i**) CRH high-speed train simulation monitoring platform; (**j**) The train driver vigilance detection in experiment.

The experimental environment consists of three parts, as shown in Figure 11a. The first part is a homemade wearable BCI model for the train driver’s EEG collection in our State Key Laboratory of Traction Power. The second part is the train driver’s EEG signal data pre-processing and driver vigilance detection model. As shown in Figure 10, it is used in the virtual driving environment in the simulation centre of the State Key Laboratory of Traction Power, Southwest Jiaotong University for our proposed method. 

Figure 11b–f illustrate the EEG collection and vigilance detection experiments in the State Key Laboratory of Traction Power, Southwest Jiaotong University. Figure 11g,h are images of the high-speed train simulator of the simulation centre of the State Key Laboratory of Traction Power, which is the only high-speed simulator in the world. Figure 11i is the operation interface of the high-speed train monitoring platform. The algorithm is implemented on a laptop computer equipped with an Intel i3 1.9-GHz CPU and 4 GB of RAM with Bluetooth for communication. Figure 11j shows a sample image frame from the experiment on train driver vigilance detection.

In the experiment, ten qualified drivers with no neurological diseases wore the wireless wearable BCI system as in Figure 11b–f, and the collected EEG signals are given in Table 5. The experiments of EEG collection and diver vigilance detection are the actual data. To avoid the traffic risks of actual fatigue driving tests, the train driver experiments in the High-Speed Rail Simulator were finished in the State Key Laboratory of Traction Power. During the entire experiment, the investigators observed and recorded the subject’s physical behaviours, yawns and inclinations of the head as the drowsiness indices in a subsequent study. The train driver’s fatigue experimental conditions are set as follows:

**Condition** **1**: (1) sleep deprivation; (2) the test time is between 4 and 6 a.m. the next day.

**Condition** **2**: (1) having a normal night’s sleep; (2) the test time is between 9 and 11 a.m. the next day.

Condition 1 is identified as drowsiness, while condition 2 is identified as alertness.

**Figure 12:**
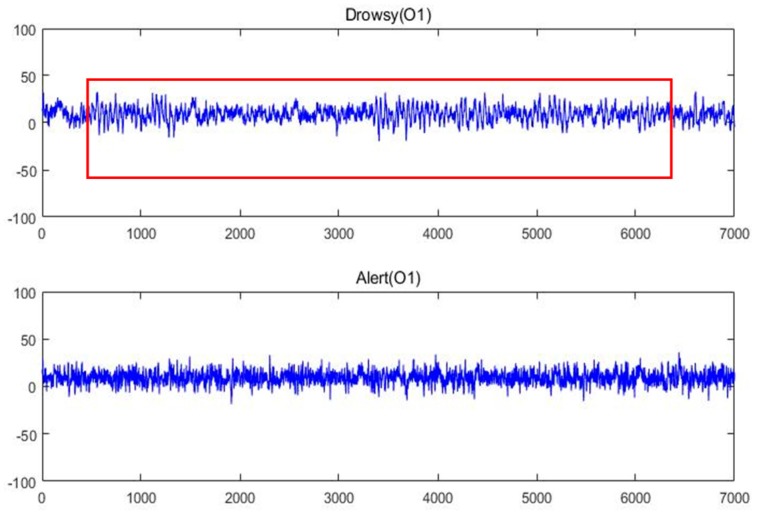
Raw EEG signal in alert and drowsy states from our equipment.

**Figure 13:**
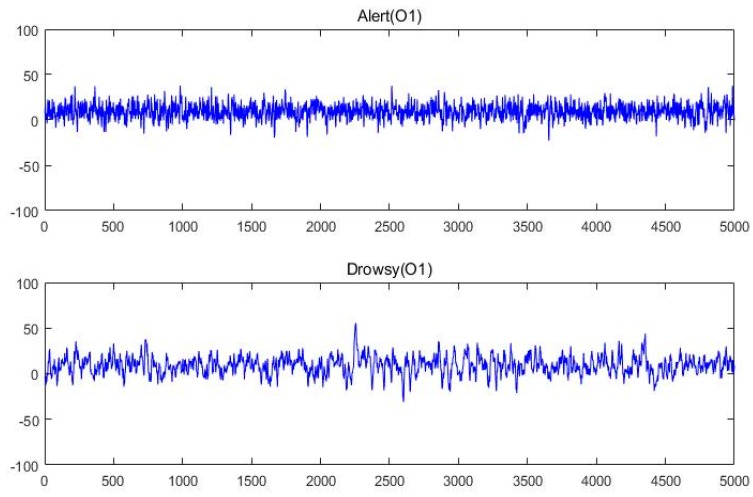
Raw EEG signal in alert and drowsy states from BP equipment.

Figure 12 shows the raw EEG data of the drowsy state and the alert state from O1, whereas Figure 13 shows the raw EEG data from BP equipment. 

**Figure 14:**
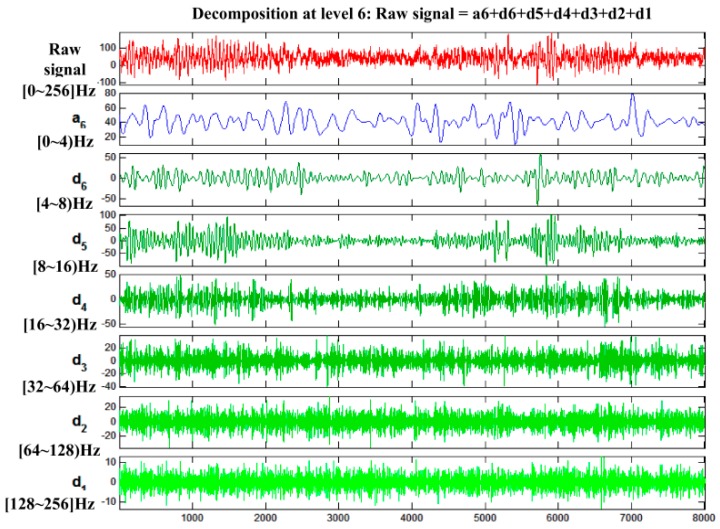
Original signal and its decomposition at each level.

The good quality of our homemade BCI system is observed. The bursting of the alpha rhythm is observed as in the red box of the drowsy state. To remove the interference, a 6-layer decomposition of the db5 wavelet is implemented in the original EEG signal. The original signal and the decomposition signal at each level and the frequency of them are shown in Figure 14. Because the hardware filter of our homemade equipment is 3–100 Hz, the signal of a6 can be regarded as noise. From Figure 14, the level **d_2_** and level **d_1_** can be regarded as noise. Therefore, by extracting the decomposition signal of **d_3_** (32–64 Hz), **d_4_** (16–32 Hz), **d_5_** (8–16 Hz), and **d_6_** (4–8 Hz), the useful EEG signal is obtained and some low- and high-frequency interferences are removed.

**Figure 15:**
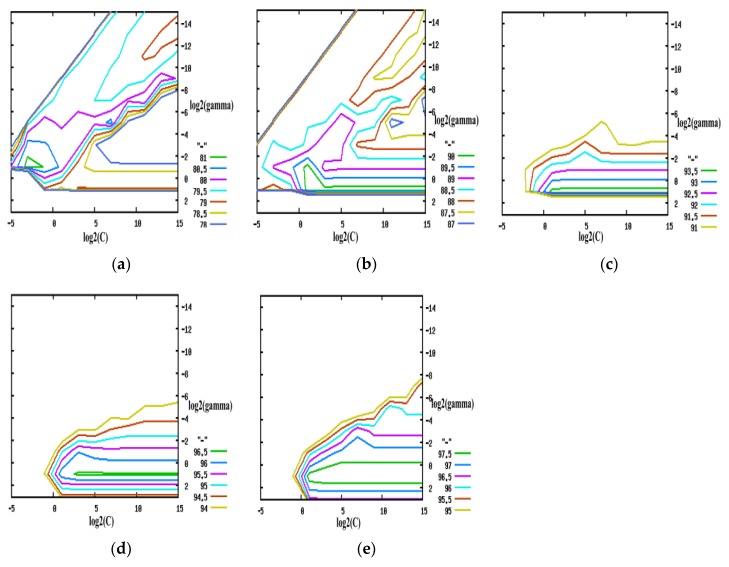
Contour line of the classification accuracy. (**a**) *r* = 0; (**b**) *r* = 1; (**c**) *r* = 2; (**d**) *r* = 3; (**e**) *r* = 4.

Libsvm-3.20, an SVM toolbox, was used in this paper to implement most functions, including data normalization, parameter optimization of the kernel function, training of the SVM classification model, and classification. Because of the excellent performance, the RBF kernel was used to map the training data to a higher-dimensional space. According to the frequency range of theta (4–8 Hz), alpha (8–14 Hz) and beta (14–34 Hz), these three frequency bands were used for feature extraction. The feature of the EEG signal at time *t* was calculated as the average PSD from time *t* − *r* to time *t*, and a Hanning window is used for feature extraction. *r* is used for feature extraction as the time window. For different *r* values, for example *r* = 0, 1, 2, 3, and 4, the contour line of classification accuracy, as shown in Figure 15, corresponds to different C (the error penalty parameter) and g (the parameter of the RBF kernel) values. This contour line provides optimal parameters of C and g, which maximize the classification accuracy, and it indicates that the optimal classification accuracy of the training data increases with the increase in *r*. To validate the proposed algorithm, cross-validation whit leave-one-out method is utilized. We dividing the original data into three parts, and two parts are considered as training data, while the remain one is considered as testing data.

This paper detects driver’s vigilance level at a fixed frequency (*r* = 0, 1, 2, 3, 4), the classification accuracy rate is defined as Equation (7):

(7) $$accuracy\;rate=\frac{correctly\;detection\;time}{total\;detection\;time}$$

Here correctly detection time presents the time which drowsiness is detected as drowsiness, while alertness is detected as alertness.

Besides, the sensitivity and the false positive are computed as Equations (8) and (9):

(8) $$Sensitivity=\frac{true\;positive}{total\;actual\;positive}$$

(9) $$False\;postive=1-\frac{true\;negative}{total\;actual\;negative}$$

Here, true positive means the time which drowsiness is detected as drowsiness, while total actual positive means the actual drowsy time. Ture negative means which alertness is detected as alertness, while total actual negative means the actual alertness time.

Table 6 and Table 7 show the average classification accuracy of O1 and O2, where drivers 1, 2, 3, 4, 5, 6, 7, 8, 9 and 10 represent different train drivers. With the increase of ***r***, the classification accuracy rate increases. As in the red box of Table 6 and Table 7, the equipment yields excellent classification efficiency, which is as high as approximately 90.70%. And as shown in Table 8, Table 9, Table 10 and Table 11, the sensitivity can be as high as approximately 86.80%, while the false positive can be down to around 5.40% when *r* = 4. In addition, the testing time of O1 and O2 as shown in Table 12 and Table 13, when *r* = 4, the minimum time is 2.16 s, which shows the good performance in real-time of this system. All these results indicate the proposed method has good performance in high-speed train drivers’ vigilance detection.

Finally, other two related studies which is also involved EEG signal are compared. One is Lin et al. [19], which proposed a BCI based smart living environmental auto-adjustment control system in UPnP home networking. They employed the Mahalanobis distance to estimate the human’s vigilance level. The other is Li et al. [20]. They propose to apply SVM based posterior probabilistic model (SVMPPM) for automated drowsiness detection. Concrete details are seen in Table 14.

## 7. Conclusions

In this paper, the design of a fatigue detection system for high-speed trains based on the train driver vigilance using wireless wearable EEG is presented. EEG collection technology, wireless wearable technology and human’s vigilance detection technology are combined for the first time to address high-speed train safety. In addition, a high-speed train driver’s fatigue warning device is designed to warn the train driver and the TCC.

The final experimental results show the validity of our method in simulation and tests. Especially, the equipment gives an excellent classification efficiency, which is as high as approximately 90.70%, when the time-window is 4. At the same time, the sensitivity can be as high as approximately 86.80%, while the false positive rate can be reduced to around 5.40%. In addition, the minimum testing time is 2.16 s, which shows the good performance in real-time of this system. All these results indicate that the proposed method has good performance in driver vigilance detection. In both theoretical analysis and practical experiments, the proposed system demonstrates the feasibility of the proposed fatigue-detecting system for high-speed train. The proposed method can be essential part of high-speed train operational safety.

## Figures and Tables

**Table 1:** Numbers and percentages of train accidents and adverse events.

Occurrence Category	Type of Adverse Event	Number	Percentage	
Collision	Human failure (HF)	1	1.28%	28.2%
	Technical failure (TF)	3	4.83%	
	External intrusion (EI)	5	6.41%	
	HF+TF	2	2.56%	
	HF+EI	9	11.5%	
	TF+EI	2	2.56%	
	HF+TF+TF	0	0	
Derailment	Human failure (HF)	2	2.56%	
	Technical failure (TF)	13	16.6%	
	External intrusion (EI)	6	7.69%	32.1%
	HF+TF	4	5.13%	
	HF+EI	0	0	
	TF+EI	0	0	
	HF+TF+TF	0	0	
Level crossing occurrence	Human failure (HF)	0	0	
	Technical failure (TF)	0	0	
	External intrusion (EI)	2	2.56%	19.2%
	HF+TF	0	0	
	HF+EI	12	15.3%	
	TF+EI	0	0	
	HF+TF+TF	1	1.28%	
Others	16	16	20.5%	
Total	78	78	100%	

**Table 2:** Numbers and percentages of train accidents based on Human failure (HF).

Occurrence Category	Number of Related Human Failures (HFs)	Percentage
Collision	12	15.38%
Derailment	6	7.69%
Level crossing occurrence	13	16.67%
Total Top 3 frequent occurrence	31	39.74%
Total cases	78	100%

**Table 3:** Railway-Performance Shaping Factors (R-PSFs) of train safety in the accident analysis.

No.	Railway-Performance Shaping Factors (R-PSFs) Total Top 10 Factors	Categories	Accidents	Percentage
1	Safety culture (SMS)	Organization	115	19.49%
2	Distraction (loss of concentration or vigilance)	Personal	93	15.76%
3	Communication-team work	Team	90	15.25%
4	System design	System	71	12.03%
5	Quality of procedures	Organization	58	9.83%
6	Perception	Personal	56	9.49%
7	Train (experience)	Personal	34	5.76%
8	Fatigue (shift pattern)	Personal and Organization	34	5.76%
9	Workload (time pressure and stress)	Task and Personal	22	3.73%
10	Quality of information	Team	17	2.88%
	Total		590	100%

**Table 4:** Survey and comparison of the existing systems used for driver vigilance monitoring.

Category	Technologies	Pros (Advantage)	Cons (Disadvantage)	Countries
Vehicle-behaviour-based technology	Using lane departure, steering wheel movements, and the pressure of the driving pedal	This technology provides a non-invasive method for the driver	It is difficult to construct a common model due to variability and changes of road circumstances. It is not suitable for high-speed trains because they use a track	America, Europe, South Korea, Japan and China
Driver-behaviour-based system	Using eye tracking, percent eye closure and the expression of the driver’s face	It provides a non-invasive method for the driver. Recent progress in machine vision and computer hardware have made it possible to measure the driver’s vigilance	Video is susceptible to driving conditions, such as light conditions. False estimation can also be caused, such as sleeping with open eyes. If the driver leaves the cockpit of train, these technologies cannot detect the driver’s behaviour	America, Europe and France
Driver-physiological-signal-based algorithm	Using electroencephalography (EEG), electrooculography (EOG), and Heart Rate Variability (HRV)	These systems are more reliable because physiological drowsiness signs are well known and rather similar from one driver to another. The EEG signal is regarded as a “gold standard” of vigilance detection. In this paper, a wireless wearable EEG signal collection system for high-speed train drivers is presented	The difficulties of the driver-physiological-signal-based measures are in how to obtain EEG signal recordings comfortably under driving conditions and classify the driver vigilance with so many EEG signals. At the same time, the wearable comfortable EEG collection system is very important for train drivers	America, Europe, Japan and China

**Table 5:** Ten drivers served in the experiment.

Driver Sum	Subject	Number	Age
10	Male	7	24
			26
			40
			42
			24
			22
			19
	Female	3	25
			24
			26

**Table 6:** Classification accuracy (%) of the testing data (O1).

*r* (s)	Driver1	Driver2	Driver3	Driver4	Driver5	Driver6	Driver7	Driver8	Driver9	Driver10
0	77.49	78.71	78.49	85.63	85.10	85.73	81.86	79.77	79.20	76.71
1	80.27	83.57	80.26	88.44	86.46	87.93	82.62	80.35	81.81	81.11
2	84.74	86.13	81.23	89.19	88.84	88.87	86.21	86.53	85.07	85.45
3	88.70	89.42	85.34	91.16	89.74	89.24	87.32	89.19	87.79	90.95
4	91.18	92.69	95.64	93.02	90.70	91.11	91.91	93.90	91.35	92.44

**Table 7:** Classification accuracy (%) of the testing data (O2).

*r* (s)	Driver1	Driver2	Driver3	Driver4	Driver5	Driver6	Driver7	Driver8	Driver9	Driver10
0	85.78	88.68	76.15	81.27	77.43	82.13	79.33	78.31	81.90	76.67
1	87.46	91.26	83.09	82.57	81.88	83.91	83.24	82.24	84.88	80.52
2	91.42	93.91	87.50	83.76	84.93	87.68	86.71	85.45	87.19	83.62
3	95.09	96.26	91.69	88.79	86.28	88.97	88.51	87.51	91.74	87.18
4	96.38	98.25	93.19	93.46	91.59	92.73	91.57	91.93	93.17	94.94

**Table 8:** Sensitivity (s) of testing data (O1).

*r* (s)	Driver1	Driver2	Driver3	Driver4	Driver5	Driver6	Driver7	Driver8	Driver9	Driver10
0	68.34	70.06	69.75	79.77	79.02	79.91	74.49	71.56	70.75	67.23
1	72.26	76.89	72.25	83.67	80.92	82.97	75.56	72.37	74.42	73.44
2	78.53	80.46	81.23	84.71	84.23	84.27	80.58	81.02	78.99	79.52
3	84.03	85.03	85.34	87.43	85.47	84.78	82.12	84.71	82.77	87.14
4	87.46	89.54	93.59	89.99	86.80	87.36	88.47	91.20	87.70	89.20

**Table 9:** Sensitivity (s) of testing data (O2).

*r* (s)	Driver1	Driver2	Driver3	Driver4	Driver5	Driver6	Driver7	Driver8	Driver9	Driver10
0	79.98	84.01	66.44	73.67	68.25	74.87	70.94	69.50	74.55	67.17
1	82.31	87.57	76.20	75.49	74.52	77.37	76.43	75.03	78.72	72.61
2	87.79	91.22	82.37	77.16	78.79	82.62	81.27	79.52	81.94	76.96
3	92.83	94.43	88.16	84.16	80.67	84.41	83.77	82.38	88.23	81.92
4	94.59	97.15	90.23	90.60	88.03	89.60	88.00	88.50	90.20	92.63

**Table 10:** False positives (s) of testing data (O1).

*r* (s)	Driver1	Driver2	Driver3	Driver4	Driver5	Driver6	Driver7	Driver8	Driver9	Driver10
0	13.34	12.64	12.77	8.51	8.83	8.45	10.77	12.02	12.35	13.81
1	11.72	9.75	11.72	6.79	8.00	7.11	10.32	11.67	10.80	11.22
2	9.05	8.20	11.15	6.33	6.55	6.53	8.16	7.96	8.85	8.62
3	6.63	6.19	8.68	5.11	5.99	6.30	7.48	6.33	7.19	5.24
4	5.10	4.16	2.31	3.95	5.40	5.15	4.65	3.40	5.00	4.32

**Table 11:** False positive (s) of testing data (O2).

*r* (s)	Driver1	Driver2	Driver3	Driver4	Driver5	Driver6	Driver7	Driver8	Driver9	Driver10
0	8.42	6.65	14.14	11.13	13.39	10.61	12.28	12.88	10.75	13.83
1	7.39	5.05	10.04	10.35	10.76	9.55	9.95	10.55	8.96	11.57
2	4.95	3.40	7.37	9.64	8.93	7.26	7.85	8.62	7.56	9.72
3	2.65	1.91	4.78	6.58	8.11	6.47	6.75	7.36	4.75	7.56
4	1.84	0.65	3.85	3.68	4.84	4.14	4.86	4.65	3.86	2.75

**Table 12:** Testing time (s) of O1.

*r* (s)	Driver1	Driver2	Driver3	Driver4	Driver5	Driver6	Driver7	Driver8	Driver9	Driver10
0	0.95	0.94	0.98	0.96	0.98	0.93	0.94	0.96	1.01	1.00
1	1.27	1.29	1.31	1.28	1.31	1.30	1.35	1.30	1.35	1.30
2	1.57	1.58	1.59	1.59	1.59	1.58	1.60	1.59	1.63	1.55
3	1.89	1.91	1.90	1.90	1.93	1.92	1.87	1.91	1.88	1.88
4	2.19	2.16	2.29	2.22	2.21	2.23	2.24	2.31	2.21	2.19

**Table 13:** Testing time (s) of O2.

*r* (s)	Driver1	Driver2	Driver3	Driver4	Driver5	Driver6	Driver7	Driver8	Driver9	Driver10
0	0.95	0.96	0.98	0.98	0.98	0.92	0.94	0.95	0.96	0.97
1	1.29	1.31	1.29	1.31	1.33	1.29	1.28	1.30	1.31	1.26
2	1.66	1.64	1.59	1.65	1.60	1.60	1.62	1.60	1.61	1.55
3	1.87	1.93	1.92	1.94	1.89	1.93	1.92	1.88	1.87	1.94
4	2.21	2.30	2.36	2.26	2.26	2.22	2.29	2.18	2.21	2.21

**Table 14:** Comparison.

Reference NO.	Preprocess	Time Window	Model	Signal Source	Terminal Device	Accuracy (%)
[19]	Band-pass filter	10 min	Mahalanobis distance	Single-Channel	-	82
[20]	Band-pass filter	1 min	SVMPPM	Three-Channel	Smartwatch	88.6
Present work	DWT	*r* (s)	SVM	Eight-Channel	Massage Chair	90.70

## Supplementary Materials

the Supplementary Materials are available online at http://www.mdpi.com/1424-8220/17/3/486/s1.
